# Next Generation Sequencing in MPNs. Lessons from the Past and Prospects for Use as Predictors of Prognosis and Treatment Responses

**DOI:** 10.3390/cancers12082194

**Published:** 2020-08-06

**Authors:** Vibe Skov

**Affiliations:** Department of Hematology, Zealand University Hospital, Vestermarksvej 7-9, 4000 Roskilde, Denmark; vihs@regionsjaelland.dk

**Keywords:** myeloproliferative neoplasms, essential thrombocythemia, polycythemia vera, myelofibrosis, next generation sequencing, gene mutations, prognosis, outcome, treatment modalities, early treatment

## Abstract

The myeloproliferative neoplasms (MPNs) are acquired hematological stem cell neoplasms characterized by driver mutations in *JAK2*, *CALR*, or *MPL*. Additive mutations may appear in predominantly epigenetic regulator, RNA splicing and signaling pathway genes. These molecular mutations are a hallmark of diagnostic, prognostic, and therapeutic assessment in patients with MPNs. Over the past decade, next generation sequencing (NGS) has identified multiple somatic mutations in MPNs and has contributed substantially to our understanding of the disease pathogenesis highlighting the role of clonal evolution in disease progression. In addition, disease prognostication has expanded from encompassing only clinical decision making to include genomics in prognostic scoring systems. Taking into account the decreasing costs and increasing speed and availability of high throughput technologies, the integration of NGS into a diagnostic, prognostic and therapeutic pipeline is within reach. In this review, these aspects will be discussed highlighting their role regarding disease outcome and treatment modalities in patients with MPNs.

## 1. Introduction

The BCR-ABL negative myeloproliferative neoplasms (MPNs) essential thrombocythemia (ET), polycythemia vera (PV), and myelofibrosis (MF) including primary myelofibrosis (PMF) and secondary myelofibrosis (SMF) (post-ET MF/post-PV MF) are characterized by uncontrolled clonal proliferation of the hematopoietic stem and progenitor cells [1]. Patients with MPNs have a huge morbidity and co-morbidity burden due to a high risk of cardiovascular and cerebrovascular complications. Chronic inflammation in MPNs is associated with increased levels of circulating cytokines and reactive oxygen species leading to genetic instability favoring clonal evolution followed by premature atherosclerosis, increased risk of thrombohemorrhagic complications, leukemic transformation, and development of second cancer [2,3,4,5,6,7,8]. 

The somatic driver mutations *JAK2V617F*, *CALR* and *MPL* are included in the diagnostic criteria for MPNs and account for the majority of cases with the remaining cases termed triple negative [9,10,11,12,13,14,15,16,17,18]. *JAK2V617F* positive MPNs may develop in a biological continuum from the early cancer stages (ET, PV) to advanced MF over decades implying an increase in the *JAK2V617F* mutational load [9,19]. In addition to the three driver mutations, acquisition of additional mutations occurs frequently in patients with MPNs [20]. 

There is a spectrum of treatment options in MPNs primarily focusing on alleviating symptom burden, reducing thrombotic complications, and targeting the malignant clone [21,22,23,24,25,26,27,28,29,30,31,32,33]. Early treatment at the time of diagnosis, where patients have the least tumor burden, has been argued to be a prerequisite to prevent clonal evolution, subclone formation, and additional mutations [27,34,35,36,37]. 

In the past decade, there has been a tremendous increase in knowledge of the complex mutational landscape in MPNs, which has revolutionized research leading to an unprecedented change in diagnosis, prognosis, classification, treatment, outcome, and response evaluation [20,38]. In the present review, past and present advances in genomics are highlighted with focus on next generation sequencing (NGS) regarding disease progression, prediction of outcome, and treatment planning in patients with chronic phase MPNs.

## 2. Genomics in MPNs

### 2.1. NGS Analysis of Somatic Gene Mutations

The dominant gain of function mutation *JAK2V617F* is present in most patients with MPNs including approximately 98% of patients with PV and 50–60% of patients with ET or PMF [39,40]. The valine to phenylalanine substitution at position 617 of the *JAK2* gene leads to constitutive activation of the JAK-STAT signaling pathway transforming hematopoietic cells to cytokine-independent growth, thereby promoting tumorigenesis, tumor progression, and inflammation [41,42]. The remaining 2% of PV patients carry somatic driver mutations in *JAK2* exon 12 [43,44]. Another driver mutation, the thrombopoietin receptor gene *MPL*, is found in up to 5 or 10% of ET or PMF patients, respectively, with *MPLW515L/K* being the most common mutation [14,15,18,45,46,47]. As the *JAK2V617F* mutation, the *MPL* mutation confers constitutive, cytokine-independent activation of the JAK-STAT pathway [14].

Somatic driver mutations in *CALR* located in exon 9 resulting in a mutant protein with a novel C-terminal were revealed by exome sequencing in 2013 in one of the first NGS studies with mutation profiling of more than one patient with MPNs [12,13]. *CALR* mutations, of which a 52 bp deletion and a 5 bp insertion are the most prevalent, are found in up to 25 or 30% of patients with ET or PMF, respectively [12,13,48]. 

Non-driver somatic mutations implicated in the disease pathogenesis of MPNs belong to various functional classes. Mutations in the epigenetic regulator genes can be divided into DNA methylation (*DNMT3A*, *IDH1*, *IDH2*, *TET2*) and chromatin modifiers (*ASXL1*, *BCOR*, *BCORL1*, *EZH2*, *KMT2A*, *KMT2C*, *KMT2D*, *SUZ12*). In addition, mutations have been observed in genes related to RNA splicing (*SF3B1*, *SRSF2*, *ZRSR2*, *U2AF1*), signaling pathway genes (*CBL*, *FLT3*, *GNAS*, *KIT*, *KRAS*, *NF1*, *NRAS*, *PTPN11*, *SH2B3*), transcription factors (*CEBPA*, *CUX1*, *ETV6*, *GATA1*, *GATA2*, *IKZF1*, *NFE2*, *NPM1*, *RUNX1*, *SETBP1*), tumor suppressors (*CDKN2A*, *NOTCH1*, *PHF6*, *RB1*, *TP53*), DNA damage response pathway (*ATM*, *PPM1D*) or cohesin complex (*STAG2*) [49,50,51,52,53,54,55,56,57]. The most frequent non-driver mutations are found in the epigenetic regulator genes *TET2*, *ASXL1*, *DNMT3A,* and *EZH2* and in the RNA splicing genes *SRSF2* and *U2AF1*. Gene mutation frequencies obtained from NGS studies are listed in Table 1 and described for the most common mutations below. 

Mutations in *TET2* occur in the entire coding sequence and result in loss of catalytic function leading to DNA hypermethylation [70,71,72]. *TET2* mutations are often seen in all disease entities with 10–20% of ET, 15–30% of PV, 10–15% of PMF patients and most frequently in patients with SMF (20–40%) [58,59,60,62,63,64,66,73]. Nonsense and frameshift mutations in exon 13 are the most common mutations in *ASXL1* resulting in loss of function and a truncated protein [74]. *ASXL1* mutations occur in 5–10% of patients with ET or PV, 20–45% of patients with PMF and 10–25% of patients with SMF [58,59,60,62,63,64,65,67,68,75]. Although most mutations in *DNMT3A* are observed in exon 23, mutations occur in the entire coding region resulting in loss of catalytic activity and altered methylation patterns [71,72,76]. *DNMT3A* mutations are most frequent in patients with PMF (5–15%) and PV (3–15%), and less observed in ET (<10%) and SMF (<5%) [58,59,60,61,62,63,64,65,77]. *EZH2* mutations are generally loss of function mutations that can be detected throughout the coding sequence and cause protein truncation [78,79]. *EZH2* mutations are primarily seen in SMF (5–15%) and PMF (3–12%), and in less than 5% of patients with ET or PV [59,61,62,63,64,65,66,68]. Finally, *U2AF1* mutations alter their 3′ splice acceptor preferences and *SRSF2* mutations result in skewed mRNA motif recognition, both mutations leading to mis-splicing of several genes [80,81]. *U2AF1* mutations are observed in 5–20% of patients with PMF or SMF and are only rarely seen in patients with ET or PV (<5%) [58,59,60,61,62,63,65,66,82,83]. Most mutations in *SRSF2* are detected in PMF (10–35%) and in less than 5% of patients with ET, PV, or SMF [58,59,60,61,62,63,66,67,68,84]. The remaining additional mutations listed above occur with frequencies lower than approximately 10% in all disease entities [20,58,59,60,61,62,63,64,65,85]. 

### 2.2. Interaction of Somatic Gene Mutations Refined by NGS Analysis 

A number of mutations co-occur frequently or are mutually exclusive in patients with MPNs. *JAK2V617F* are frequently associated with mutations in the epigenetic regulator genes, *ASXL1* [63,64,67,86,87,88], *DNMT3A* [61,63,86], *EZH2* [64,67,87,88,89], *IDH2* [61,64,67,86], or *TET2* [63,64,86,87,88] and to a lesser extent with mutations in *CBL* [86,89], *CUX1* [88], *IDH1* [67,86], *NOTCH1* [61], *NRAS* [86,89], *RUNX1* [88], *SF3B1* [86,88,89], *SETBP1* [88], *SH2B3* [86], *SRSF2* [67,89], *TP53* [89], *U2AF1* [60,89], or *ZRSF2* [88,90]. Mutations in *CALR* are reported to co-occur with *ASXL1* [67,83,88,91], *DNMT3A* [64,89], *TET2* [75,77,89,91], or *U2AF1* [83,90,91] and less with *EZH2* [91], *IDH2* [75,83], *NRAS* [91], *RUNX1* [90], *SF3B1* [91], *SRSF2* [83,90], or *TP53* [75,91]. Mutations in *MPL* are found associated with *ASXL1* [67] or *SRSF2* [60]. 

Mutations in epigenetic regulator genes result in deregulated gene expression, aberrant cell function and disease, and these genes are often co-mutated with each other or with mutations in other functional classes such as RNA splicing, signaling pathways or transcription factors. Focusing on non-driver somatic mutations, a number of NGS studies have shown a frequent co-existence of mutations in *ASXL1* with *EZH2* [20,59,61,62] or *U2AF1* [13,20,60,62], and less often with *DNMT3A* [64], *IDH2* [59], *RUNX1*, *KIT* [59], *CBL* [20,60], *SRSF2*, *NRAS* [20], or *SETBP1* [60]. Although *ASXL1* and *EZH2* are related to the PRC2 complex, they are not mutually exclusive [92,93,94]. Another frequently mutated epigenetic regulator gene in MPNs, *TET2* is not as frequently co-mutated as *ASXL1* and is found associated with *SRSF2* [13], *SH2B3* [59], *SUZ12*, *CBL*, or *PTPN11* [60]. Other NGS studies showed concomitant mutations in *IDH1/2* with *SRSF2* [13,60,89] or *U2AF1* [89], *U2AF1* with *PTPN11* [60] or *CBL* [62], *EZH2* with *SETBP1* or *CBL* [62], and *NRAS* with *SETBP1* or *NF1* [62]. 

The two epigenetic regulator genes *IDH1/2* and *TET2* are almost never co-mutated but mutually exclusive and share the same functional mechanisms by altering the hypermethylation signature of hematopoietic cells. IDH enzymes normally produce α-ketoglutarate (αKG), however, cancer cells with mutated *IDH1/2* produce 2-hydroxyglutarate (2-HG) which is detrimental to *TET2*, because *TET2* uses αKG as a co-activator, resulting in *TET2* having impaired functionality [95]. Importantly, mutations in *IDH1/2* or *TET2* are not equally distributed across disease entities in that IDH1/2 are more frequent in blast phase disease whereas *TET2* occurs with equal frequency in chronic and blast phase disease [54]. 

Driver mutations are in most cases mutually exclusive, however, patients with two driver mutations have been observed. *JAK2V617F* mutations have been reported to co-exist with *MPL* mutations in all three disease entities [15,96,97,98] or with *CALR* mutations [99,100]. In a targeted NGS study of 1 patient with ET, *JAK2V617F* and *MPLW515* were co-mutated, and interestingly, these mutations were not found by standard molecular screening [101]. 

Non-driver and driver mutations in signaling pathway genes are generally mutually exclusive, but may co-occur and then usually in different clones [54]. Concomitant mutations in the signaling pathway genes *JAK2V617F* and *SH2B3* have been demonstrated in blast-phase MPN and more rarely in chronic phase disease [102]. With rare exceptions, spliceosome mutations are mutually exclusive of each other and lead to abnormal splicing, exon skipping and impaired hematopoiesis [53,103]. This is in accordance with a NGS study of 182 patients with PMF by Tefferi et al., who found that *SRSF2* rarely coexists with *U2AF1* [60]. 

## 3. NGS in the Diagnosis and Prognosis of MPNs

### 3.1. Development and Progression of Somatic Gene Mutations in Patients with MPNs

Development of disease processes in hematological neoplasms may usually begin several years before clinical manifestation [104]. Mutations in MPNs arise in the hematopoietic stem and progenitor cells (HSPCs) in the bone marrow, where a number of cells may acquire somatic mutations that pass on to the next generation of cells [105,106]. Several of these mutations have a benign effect or are deleterious and will become extinct, however, few mutations increase proliferation and fitness of the cells resulting in increased clonal expansion [107,108]. This process is named Clonal Hematopoiesis of Indeterminate Potential (CHIP) defined by the presence of somatic mutations with an allele burden of more than 2%, but without presence of a hematological abnormality or malignancy [109]. This premalignant stage may be characterized by one or more somatic mutations in genes associated with hematological neoplasms, such as predominantly *TET2*, *DNMT3A*, *ASXL1*, but also *JAK2*, *SF3B1*, *SRSF2*, *TP53* or *PPM1D* [104,110,111,112,113] and is present in 10–20% of healthy, older individuals over age 70, but only in 1% of healthy individuals below age 50 [104,111]. Interestingly, one study of 3067 blood donors aged 17–70 and 1152 unselected individuals aged 60–98 years only observed the spliceosome mutations *SF3B1* and *SRSF2* in those aged over 70 years suggesting that these clones expand later in life [114]. Jaiswal et al. found a 50 fold higher risk of developing a hematological malignancy in individuals with a mutant allele burden > 10% [111], indicating the importance of variant allele frequency assessment in prognostication and prediction of disease progression. 

Autoimmune diseases, smoking, chemotherapy, and chronic inflammation may impose a higher selective pressure on the HSPC pool with a more rapid outgrowth of mutant clones, thereby facilitating the development of CHIP. This inflammatory environment leads to impaired fitness, accelerated aging, and exhaustion of the HSPC pool [115,116,117,118,119]. Individuals with CHIP have a higher risk of developing a myeloid neoplasm, but are also at higher risk of developing cardiovascular diseases, type 2 diabetes, or second cancer and have a higher all-cause mortality [105,107,111,119]. Clonal expansion of the founding clone from CHIP towards a hematological neoplasm may include linear acquisition of cooperating mutations yielding subclone formation or mutations that develop in parallel to the founding clone [120] together with an increasing variant allele frequency [111]. These mutations may include *JAK2*, *CALR* or *MPL* [113,121]. In a NGS study of 197 patients with MPNs, Lundberg et al. presented a model of clonal evolution from CHIP to initiation of MPNs showing that most initial mutational hits occur in *JAK2V617F* or *CALR* or in the epigenetic regulator genes *DNMT3A* or *TET2* [120]. Although NGS studies using DNA from colony formation assays or monitoring of the mutant allele burden enable assessment of the clonal hierarchy, single cell sequencing is a great tool to unveil the signature of genetically distinct subclones during clonal evolution and disease development. In the first single cell exome sequencing study in MPNs performed by Hou et al. in 2012, principal component analysis and mathematical modeling of data from 58 cells from a *JAK2V617F* negative patient with ET indicated monoclonal evolution of the disease [122]. 

#### 3.1.1. From CHIP to More Advanced Stages of MPNs

Non-driver mutations may chronologically precede or follow the acquisition of driver mutations in the clonal evolution from CHIP to early and blast phase MPN. Lundberg et al. performed NGS on 197 patients with MPNs and showed that mutations in *TET2* or *DNMT3A* were often present in early founding clones acquired before *JAK2V617F*, while mutations in *ASXL1*, *EZH2*, or *IDH1/2* were often acquired after *JAK2V617F* [120]. Similarly, in a comprehensive NGS study of 2035 patients with MPNs, Grinfeld et al. determined the relative probability of a gene to occur first or second in a gene pair relative to *JAK2V617F*. *DNMT3A*, *SH2B3*, *SF3B1,* or *CUX1* most likely occurred first, whereas genes such as *ASXL1*, *IDH1/2*, *NFE2*, *EZH2*, *NRAS*, *TET2*, *TP53*, or *PPM1D* most likely occurred second [20]. In contrast to *JAK2V167F*, mutations in *CALR* are suggested to be early events with additional mutations being secondary events [12,13].

The transformation of early phase MPN to more advanced stages and ultimately acute myeloid leukemia (AML) is usually associated with clonal expansion and acquisition of further mutations. Mutations in *ASXL1*, *EZH2*, *IDH1/2*, *SRSF2*, or *TET2* may frequently occur in the MF transformation phase, and *RUNX1* or *TP53* in the transformation phase to AML [62,121]. A study using whole genome and capture sequencing provided evidence for clonal expansion in one patient with PMF transforming to AML. Based on variant allele frequencies, they revealed a founding clone in *JAK2V617F* or *U2AF1* and three subclones with the *MYB* subclone developing parallel to the nested subclones harboring *ASXL1/HCFC1* and *RUNX1/IDH1*, the latter expanding during transformation to AML [123]. A low or an increasing allele frequency in an early event mutation such as *DNMT3A* may persist for years without signs of hematological disease [104]; however, an increasing allele frequency with loss of the wild type allele in a late event mutation—*TP53*—usually have a deleterious effect leading to rapid clonal expansion and AML [120]. 

Another NGS study of 50 *JAK2V617F* positive patients with ET or PV demonstrated a decrease in the *JAK2V617F* allele burden during 3 years of follow-up in parallel with an increasing allele burden of other mutations in two patients suggesting clonal competition. However, 12 out of the 24 patients with disease progression were treated with hydroxyurea, which accordingly also might have impacted the decrease in the *JAK2V617F* allele burden [86]. Although increasing allele frequency of the *JAK2V617F* mutation has been associated with fibrotic progression or thrombosis in patients with MPNs [124,125,126], low *JAK2V617F* allele burden has been observed in PMF transforming to blast phase [127,128,129]. These findings indicate progression of the disease in line with other reports showing that leukemic transformation in *JAK2V617F* positive MPNs usually occur in *JAK2V617F* wild type cells [130,131]. Furthermore, low *JAK2V617F* allele burden might be related to a possible co-occurring mutation in *MPL* [97], however, a study showed that low *JAK2V617F* allele burden was associated with poor survival without any co-occurring *MPL* mutation [128] suggesting the presence of an overriding malignant *JAK2V617F* negative subclone [127], calling for NGS studies to be performed upfront to reveal these co-occurrences. Interestingly, in their NGS study of 2035 patients with MPNs, Grinfeld and colleagues found that clone size for most genes has no impact on outcome suggesting that the most aggressive subclone determined outcome [20].

#### 3.1.2. Implication of Mutation Order on MPN Phenotype

The temporal order of acquisition of mutations in *JAK2V617F* and epigenetic regulator genes influences the phenotypic presentation of the MPN disease. In an NGS study from 2015, Ortmann and colleagues described the order of mutations in 48 *JAK2V617F* and *TET2* mutated patients with MPNs. Among *JAK2*-first patients, there was an overrepresentation of homozygous PV patients at younger age having a higher risk of thrombotic events, while *TET2*-first patients were common among both ET and heterozygous PV patients at older age [132]. Later the same year, Nangalia et al. showed that seven of 10 *JAK2*-first patients presented with PV and six of 6 *DNMT3A*-first patients with ET, however, they found no association between *DNMT3A*-first and age or thrombotic risk [133]. In agreement, the NGS study of 2035 patients with MPNs by Grinfeld et al. showed that *JAK2V617F* was more likely an early event in patients with PV or MF and a secondary event in those with ET [20]. Stratified by mutation type, the authors found that *DNMT3A*, *TET2*, *ASXL1,* or *EZH2* mutations occurred more often first in patients with ET compared to patients with PV or MF, however, *TET2* or *DNMT3A* were more likely also an early event in patients with MF [20]. 

**Figure 1:**
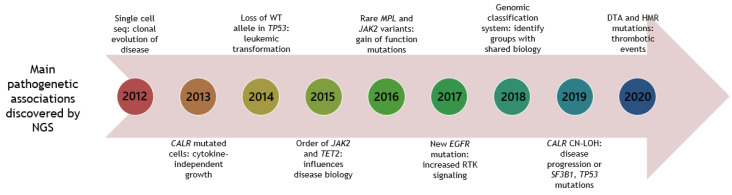
Timeline of NGS studies in patients with MPNs, unravelling main pathogenetic associations. Year is shown in circular boxes. Year 2020 is until June 30. CN-LOH: copy neutral-loss of heterozygosity. DTA: *DNMT3A*, *TET2*, *ASXL1*. HMR: high-molecular risk mutations (*ASXL1*, *EZH2*, *IDH1/2*, *SRSF2*). RTK: receptor tyrosine kinase.

In Figure 1, the main pathogenetic associations discovered by NGS are depicted.

With the advent of NGS technologies and its increasing use in clinical routine, the identification of CHIP associated mutations has been possible and may support clinical decision making in MPNs. In addition, NGS may be applied to follow clonal expansion, thereby identifying individuals at risk of developing a hematological malignancy or progressing to advanced stages of the disease.

### 3.2. NGS in the Diagnostic Decision-Making in MPNs 

According to the revised 2016 WHO criteria, in triple-negative patients, testing for the most frequent additional mutations (e.g., *ASXL1*, *DNMT3A*, *TET2*, *EZH2*, *IDH1/2*, *SRSF2*) may be helpful to determine the clonal nature of the disease and complement the morphological criteria [38,65,134]. In 2018, the European Leukemia Network expert panel recommended that testing for mutations in the “high molecular risk” (HMR) mutations *ASXL1*, *EZH2*, *IDH1/2* and *SRSF2* should be performed in patients negative for the driver mutations in *JAK2*, *CALR,* or *MPL*. Although only testing for the 5 HMR mutations in triple-negative MF was recommended, testing for these mutations in triple-negative ET and for *TP53*, *TET2*, *DNMT3A*, and *CBL* in both disease entities was a matter of debate and may be performed by each institutions own preference [135]. Another advantage of using NGS for mutation detection in triple-negative MPNs is the possibility of simultaneous testing of rare variants in *JAK2*, *CALR,* or *MPL* otherwise not detected by conventional assays.

At the time of diagnosis of MPNs, single gene analyses of the driver mutations in *JAK2*, *CALR,* or *MPL* have been performed for more than a decade by conventional molecular biological tools. However, with NGS becoming increasingly implemented in the diagnostic laboratories, the application of NGS for concomitant detection of driver and non-driver mutations at the time of diagnosis for all patients suspected for MPNs may likely be routine in the years to come. Distinction between MPN subtypes is challenging and relies on clinical and morphological criteria. Besides the type of variant, studies have shown that the number [13,58,65,66] and order of mutations [20,132,133] may affect the phenotype, and thereby the specific subtype of the disease. Interestingly, classification of MPN subtypes has been performed in the NGS study by Grinfeld et al. Based on clinical variables, germline, and somatic mutations, they were able to predict if patients were diagnosed with ET or PV, or with chronic phase disease or MF [20]. Thus, upfront application of NGS can assist in confirming the diagnosis and may allow for simultaneous assessment of the molecular complexity of the disease. With increased coverage and sensitivity as well as lower costs, it seems likely that NGS will be part of the diagnostic testing algorithm in most laboratories in the near future.

### 3.3. Implication of Somatic Gene Mutations on Prognosis, Risk Stratification and Outcome Revealed by NGS

The genomic landscape of patients with MPNs is highly complex with mutations in both driver and non-driver genes conferring an increased risk of disease progression and transformation to AML affecting prognosis, molecular risk stratification, and outcome. For nearly a decade, several NGS studies have been performed in patients with MPNs providing a thorough investigation of the implication of gene mutations on these aspects. In the following, these studies will be reviewed in more detail with focus on non-driver mutations. In Table 2, the majority of NGS studies conducted from 2012–2020 in patients with MPNs is listed.

The first NGS studies on more than one patient with MPNs appeared simultaneously in 2013, when Nangalia et al. and Klampfl et al. described the new *CALR* mutations in two separate studies [12,13]. In their series of 151 patients with MPNs, Nangalia et al. included 62 patients with ET and provided evidence for a significantly higher rate of transformation to MF, higher platelet counts, and lower hemoglobin levels in *CALR* mutated ET patients compared to those with *JAK2V617F* [13]. Similar results were reported in follow-up studies of 176 and 89 *CALR* mutated patients with ET [136,137], however, although no evidence of a higher transformation rate was found in these studies, Grinfeld et al. found an increased risk of transformation in their NGS study of 2035 MPN patients of whom 1321 had ET [20]. Accordingly, the higher power of the NGS studies by Grinfeld et al. and Nangalia et al. may account for the differences in risk of transformation. The study by Klampfl et al., which was mainly performed by Sanger sequencing and fragment analysis, included 311 ET patients and 203 patients with PMF showing higher platelet counts and lower leukocyte levels in *CALR* mutated patients in both cohorts together with lower hemoglobin in ET. In addition, *CALR* mutated ET patients have longer overall survival (OS) and a lower risk of thrombosis compared to *JAK2V617F* positive patients, while *CALR* mutated PMF patients have longer OS compared with both *JAK2V617F* and *MPL* positive patients [12]. These findings have been confirmed in later studies [137,138,139,140]. However, a study of 139 *CALR* type 1 mutated patients with MF demonstrate that additional mutations in *ASXL1*, *EZH2*, *IDH1/2* or *SRSF2* influenced survival and disease outcome [83]. 

#### 3.3.1. High Molecular Risk Mutations

Several studies have provided evidence for the adverse impact of somatic mutations in *ASXL1*, *EZH2*, *IDH1/2*, and *SRSF2* on shorter OS or leukemia-free survival in patients with PMF. The first simultaneous analysis of these five mutations was described in 2013 by Vannucchi et al. using Sanger sequencing in a series of 879 patients with PMF. They reported the significance of these mutations in regard to premature death and leukemic transformation and suggested them to be included in future studies [187]. In 2014, Guglielmelli et al. reported results from a NGS study of 167 patients with MF highlighting the detrimental impact of mutations in *ASXL1*, *EZH2*, *IDH1/2 or SRSF2*. In MF, a new molecular prognostic classification was for the first time proposed, where patients having a mutation in any of these five genes were classified as an HMR group [138]. These findings were confirmed by the same group in 2018 in a large NGS study of 805 patients ≤70 years with PMF [68]. Other NGS studies have shown conflicting results regarding HMR mutations. In a series of 165 patients with PET-MF and 194 patients with PPV-MF, Rotunno et al. only provided evidence for a detrimental effect of *SRSF2* in patients with PET-MF but not in PPV-MF. Surprisingly, there was no difference in outcome regarding the remaining 4 HMR mutations. The authors suggested that the mutational events occurring in PMF are different from those implicated in the transformation to SMF [67], although, Spiegel et al. found an association between HMR mutations and shorter OS in 100 patients with MF [82]. However, as opposed to the study by Rotunno et al., the study by Spiegel et al. was performed on both PMF and SMF patients, possibly accounting for the discrepancies. Nevertheless, Tamari et al. found no association between HMR mutations and OS in their NGS study of 100 patients with PMF or SMF [176]. Finally, and not in agreement with the study by Rotunno et al., Courtier at al reported an association between mutations in *SRSF2* and shorter OS in patients with PMF but not in SMF [62]. These results may support the suggestion that SMF and PMF are not two separate disease entities but should be considered as one disease entity in the biological MPN continuum, however, this is a matter of debate. Although HMR mutations have predicted poor prognosis in MF, these mutations have also been associated with poor prognosis in patients with ET and PV. In an NGS study of 50 patients with ET and PV by Luque Paz et al., patients with at least one HMR mutation at diagnosis or an increasing allele burden (relative increase of at least 20%) of at least one additional gene mutation showed disease progression after 3 years [86]. 

Individually, HMR mutations, in particular *ASXL1* or *SRSF2*, have been related to inferior outcome in predominantly patients with MF but also in patients with ET or PV [94,188,189,190,191]. Several NGS studies observed shorter OS or leukemia-free survival in MF patients with mutations in *ASXL1* [60,62,165], *SRSF2* [60,62,88,165], or *EZH2* [64,82]. Intriguingly, *CALR* type 1 mutations in PMF are not only related to longer OS but may also ameliorate the poor prognosis of *ASXL1* and *SRSF2* mutations [192,193]. In particular, PMF patients with *CALR*¯/*ASXL1*^+^ mutational status have an inferior survival [192]. In NGS studies of patients with ET or PV, inferior OS or higher risk of leukemic transformation have been associated with mutations in *ASXL1* [63,146,164], *SRSF2* [146,164], *IDH1/2* [164], or *EZH2* (only ET) [186]. However, in a single gene study of 107 ET patients with *ASXL1* mutations, they found no impact of *ASXL1* on OS [194]. In Table 3, the clinical significance of somatic mutations in MPNs refined by NGS is reported. 

#### 3.3.2. Other Groups of Adverse Mutations

Other groups of mutations have been proposed in the prognostication of patients with MPNs. In an NGS study from 2016 by Tefferi et al. of 182 patients with PMF, overall survival was reduced in *ASXL1*, *SRSF2*, *CBL*, and *KIT* mutated cases and leukemia-free survival was reduced in patients with mutations in *SRSF2*, *RUNX1*, *CEBPA*, and *SH2B3*. Accordingly, these observations led to the reporting of *ASXL1*, *SRSF2*, *CBL*, *KIT*, *RUNX1*, *CEBPA*, and *SH2B3* as an adverse group of mutations associated with inferior OS and leukemia-free survival regardless of *JAK2*, *CALR,* or *MPL* mutation status [60]. In addition, mutations in *U2AF1* were associated with anemia and thrombocytopenia, and *SRSF2* with anemia [60]. Interestingly, Tefferi et al. reported shorter OS of PMF patients with the *U2AF1Q157* mutation compared to *U2AF1S34* mutated patients or *U2AF1* unmutated patients [195] leading to the inclusion of the *U2AF1Q157* mutation as an HMR mutation in their GIPSS prognostic model in patients with PMF [165]. Besides their NGS study of patients with PMF, Tefferi et al. reported the prognosis of adverse mutations in an NGS study also from 2016 of 183 patients with ET and 133 patients with PV from the Mayo clinic followed by validation in an Italian cohort of 174 ET patients and 215 PV patients. In ET, *IDH2* and *SH2B3* were associated with inferior OS, *EZH2* and *TP53* with shorter leukemia-free survival, and *SF3B1* and *U2AF1* with shorter myelofibrosis-free survival. In PV, *ASXL1* and *SRSF2* were associated with inferior OS, *SRSF2* and *IDH2* with shorter leukemia-free survival and *SRSF2* with shorter myelofibrosis-free survival. Based on these observations, *SH2B3*, *SF3B1*, *U2AF1*, *TP53*, *IDH2*, and *EZH2* were included as adverse mutations in ET and *ASXL1*, *SRSF2*, and *IDH2* as adverse mutations in PV. Presence of at least one of these adverse mutations compared with other mutations or no mutations was associated with reduced OS, shorter leukemia-free survival and myelofibrosis-free survival in both disease entities [59]. Recently, the same group presented another NGS study also including patients from the Mayo and Italian cohorts, in total 502 ET and 404 PV patients. The authors developed a mutation enhanced prognostic system consisting of adverse mutations including *SF3B1*, *SRSF2*, *TP53*, and *U2AF1* in ET and *SRSF2* in PV affecting OS, leukemia-free survival or myelofibrosis-free survival [186]. Thus, the authors confirmed the prognostic relevance of mutations in *SF3B1*, *TP53*, and *U2AF1* in ET and *SRSF2* in PV [59,186]. In a very recent NGS study of 464 patients with MF, Coltro et al. provided evidence for an association of mutations in the RAS pathway genes *CBL*, *KRAS*, and *NRAS* with shorter OS and leukemia-free survival [57].

#### 3.3.3. Fibrotic Progression 

In 2018 and 2020, two NGS studies by Bartels et al. reported the implication of fibrotic progression in 64 patients with PV and 104 patients with prefibrotic PMF, respectively. In the study of PV patients, they observed a higher risk of fibrotic progression in patients with additive mutations such as *DNMT3A*, *IDH2*, *SRSF2*, or *U2AF1*, however mutations in *TET2* were not implicated in disease progression [89]. In prefibrotic PMF, they highlighted that mutations in *SRSF2*, *U2AF1*, *SF3B1*, *IDH1/2*, or *EZH2* in the pre-fibrotic stage were independent risk factors for rapid fibrotic progression. Although mutations in *ASXL1* are risk factors when acquired during disease progression, mutations in *ASXL1*, *DNMT3A,* or *TET2* in prefibrotic PMF were not associated with development of fibrosis [84] unless acquired after the driver mutation (*ASXL1* only). Of note, the follow-up time was only three years possibly accounting for the lacking association. Interestingly, in both studies, the allele burden of driver mutations was not associated with disease progression [84,89].

#### 3.3.4. *TET2* Mutations and Order of Mutations

The prognostic impact of *TET2* is debated and conflicting results exists [86,196,197]. The first NGS study reporting the implication of *TET2* mutations in MPNs appeared in 2014, when Lundberg et al. provided evidence for shorter OS and increased risk of leukemic transformation in 23 *TET2* mutated MPNs [120]. In the NGS study by Tefferi et al., they showed an association between *TET2* mutations and thrombosis in patients with ET independently of both age and driver mutation [59]. Interestingly, Segura-Diaz performed a targeted case-control study of 55 age-matched patients with PV and provided evidence for mutations in *TET2* and higher risk of cardiovascular disease and thrombotic events [185]. However, in two other NGS studies, no influence of *TET2* mutations on OS in patients with MF [64] or disease progression in ET or PV was observed [86]. Recently, Kralovics et al. performed targeted NGS on 163 patients from their Proud-PV cohort receiving ropeginterferon alpha-2b [182]. They found a higher baseline *JAK2V617F* allele burden in *TET2* mutated patients compared to *TET2* wild type, although statistical significance was not reached (*p* < 0.09). Nevertheless, ET and PV patients with the *TET2* mutation had a significantly higher baseline *JAK2V617F* allele burden compared with *TET2* wild type cases as reported in the serial single gene sequencing study of 40 ET and 43 PV *JAK2V617F* positive patients performed by Quintas-Cardama et al. [198]. These results suggest a more adverse prognosis of *TET2* mutated patients with ET or PV. 

In the NGS study by Ortmann et al. in 2015, the order of acquisition of *JAK2V617F* and *TET2* was comprehensively investigated in two different cohorts of patients with MPNs. The first cohort included 246 patients and the follow-up cohort 918 patients. In total, 48 patients presented with mutations in both *JAK2V617F* and *TET2*. As previously noted, although *JAK2V617F* first patients were predominantly PV patients of younger age, they have a higher risk of thrombotic events and present with abnormal blood counts compared with *TET2* first patients [132]. Furthermore, the transcriptional consequence of the *JAK2V617F* mutation in *TET2* first cells revealed increased proliferation of hematopoietic stem and progenitor cells. It was concluded that the order of *JAK2V617F* and *TET2* mutations might influence the acquisition of additional mutations and the impact of *JAK2V617F* on the proliferation rate, thereby affecting disease pathogenesis [132]. Thus, it seems likely that the conflicting results may be attributed to the time point of *TET2* acquisition.

#### 3.3.5. *TP53* and *PPM1D* Mutations

Mutations in *TP53* are rare during the chronic phase of MPNs [120] but increases rapidly during leukemic transformation [162,199]. In accordance, Lundberg et al. found in their NGS study a particular unfavorable impact of acquisition of *TP53* mutations on leukemic transformation and OS [120] also observed by Grinfeld et al. in their large NGS study [20]. Similarly, in patients with MF, *TP53* mutations were reported to result in shorter OS in three NGS studies [61,62,88]. In 254 chronic phase MPNs treated with cytoreductive drugs, Kubesova et al. found no association between low burden *TP53* mutations and leukemic transformation suggesting other factors such as genomic instability or hematopoietic exhaustion may lead to clonal expansion and leukemic transformation [162]. 

In their large series of 2035 patients with MPNs, Grinfeld et al. observed that *PPM1D* was the eighth most mutated gene. *PPM1D* is a known regulator of p53 and has been associated with the development of other cancers [200,201]. It may be speculated that MPN patients with *PPM1D* mutations may be more prone to development of second cancer. Importantly, several other MPN-associated mutations such as *ASXL1*, *SH2B3*, *TET2*, *JAK2*, *TP53*, *KRAS*, *NRAS*, and *U2AF1* are found in other cancers as well [92,202,203,204,205]. Indeed, studies have shown that patients with MPNs have a higher risk of developing second cancer [206,207,208]. 

#### 3.3.6. Prognostic Genomic Classification Models

In the study by Grinfeld et al. addressed above, the authors presented a prognostic genomic classification model applied on the 2035 patients and validated on an external cohort of 270 MPN patients [20]. Although prognostic predictive scoring systems such as IPSS [209], DIPSS [210], MIPSS [68], GIPSS [165] and MYSEC-PM [211] have been developed in the past, only patients with MF were included. Integrating 63 clinical, demographic, cytogenetic and genomic features, the authors identified eight different genomic subgroups enabling personalized prediction of outcome in patients with ET, PV, and MF [20]. The first group was characterized by *TP53* mutations or aneuploidy and a dismal prognosis, and the second by mutations in one or more of 18 myeloid genes especially spliceosome, epigenetic, or RAS genes with increased risk of disease progression or death. Patients not belonging to one of these two groups were classified according to their driver mutation either *CALR* and chr20q-, *MPL* with higher risk of AML transformation, homozygous *JAK2* or *NFE2* mutations with increased risk of MF transformation, or a heterozygous *JAK2* mutation mostly with favorable outcome. The seventh subgroup comprised other clonal mutations, and the eight included patients with no mutations and predominantly a benign outcome [20]. The authors demonstrate a model with good risk prediction that correlated well with outcome. Thus, these data show that including genomic data in clinical decision-making may improve prognostic models in MPNs. 

#### 3.3.7. Additional Mutations in Relation to Sex

Although male and female sex have a significant impact on cancer prognosis and treatment response, sex-related molecular signatures have rarely been investigated in cancer patients [212]. Recently, in a series of 227 patients with MPNs, Karantanos et al. found a higher number of additional mutations in men compared with women. Moreover, an increase of >0.5% per year of the *JAK2V617F* allele burden compared with <0.5% per year was associated with shorter OS in females, which was not observed in men. The authors concluded that male sex in patients with MPNs is an independent prognostic risk factor for poor outcome caused by an increased number of non-driver mutations, especially HMR mutations including *U2AF1* [181].

#### 3.3.8. Co-Occurring Non-Driver Somatic Mutations in Prognostication

Despite the prognostic impact of co-occurring non-driver mutations has been described in AML [213], it has only been sparsely investigated in MPNs, which may be attributed to the lower number of mutations in MPNs compared with AML. In a Sanger sequencing study, Lasho et al. reported a significant clustering of mutations in *SRSF2* with *IDH1/2* mutations, however, the prognostic relevance of mutations in *SRSF2* was independent of *IDH1/2* mutations [190]. One single NGS study showed a higher occurrence of comutated non-driver mutations in *JAK2V617F* positive ET or PV patients with progressive disease compared to patients without. However, the specific type of co-occurring variants regarding their prognostic implication was not reported [86]. Accordingly, larger studies investigating whether the type of co-occurring additional mutations may affect prognosis and outcome are warranted in the future.

#### 3.3.9. Impact of the Number of Mutations on Prognosis and Outcome

In accordance with the biological continuum from chronic phase ET and PV to the more advanced and critical stages of PMF or SMF, several NGS studies have found a higher number of mutations in MF patients compared to patients with ET or PV. In their exome sequencing study, Nangalia et al. found a significantly higher median number of mutations in patients with PMF (13.0) compared to ET (6.5) and PV (6.5) [13]. Similarly, although in lower numbers owing to a smaller amount of analyzed genes, in a targeted NGS study of 40 patients with ET, 30 with PV, and 30 with PMF, patients with PMF had an overall mean number of 2.5 mutations/patient, patients with PV 1.63, and patients with ET 1.38 mutations/patient [58]. In line with the biological continuum from chronic phase MPN to the inferior stage of post-MPN AML, Alduaij et al. reported in their targeted NGS study a median number of 1 mutation/patient in ET/PV, 2 in MF, and 4 in post-MPN AML [65].

An association between outcome and number of mutations has been demonstrated in several targeted NGS studies and in one exome sequencing study. Lundberg et al. showed in their exome sequencing study of 197 MPN patients that 2 or more mutations were associated with significantly increased risk of transformation to AML and reduced OS [120]. In agreement, two targeted NGS studies demonstrated shorter OS in patients with PMF or SMF having three or more mutations [64,82], in line with the study of 9 patients with SMF and 21 patients with PMF by Silver et al., who reported an association between adverse events and three or more mutations [157]. Likewise, in a serial single gene sequencing study of a cohort of 797 patients with PMF from Europe and Mayo clinic, Guglielmelli et al. provided evidence for a significantly shorter leukemia-free survival and OS in both cohorts in patients with two or more HMR mutations compared to one or no HMR mutations [214]. Interestingly, in their NGS study of 100 patients with SMF or PMF, Spiegel et al. observed an HMR mutation in 24% of patients with 0 to 2 mutations in contrast to 79% of patients with 3 or more mutations, the latter group having reduced OS [82]. 

Although Acha et al. demonstrated shorter OS in triple negative patients with ET or PMF having only one or more mutations, which might be attributed to the detrimental prognosis of triple negative patients with MPNs, Tefferi et al. also provided evidence for an association between reduced OS and one or more mutational hits in a cohort of *JAK2V617F* positive and negative PMF patients [60,167]. In patients with ET or PV, Tefferi et al. in their NGS study found that the number of genes is not detrimental to outcome unless specific prognostic adverse mutations are involved [59]. However, Luque Paz et al. reported shorter OS in their study of 190 ET patients with one or more mutations [170], while Andreasson et al. demonstrated shorter OS in a study of 85 PV patients with more than three mutations [63]. Accordingly, the number of mutations are of high importance in the prognostic assessment of all MPN subgroups and may be used as a risk factor in treatment planning decisions. 

#### 3.3.10. NGS and Transplantation Outcome 

Patients with adverse mutations may be candidates for hematopoietic stem cell transplantation (HCT). In DIPSS intermediate risk patients, Alduaij et al. proposed early HCT in patients with an HMR profile and delayed HCT in patients with absence of an HMR profile [65]. Nevertheless, in a series of 101 patients with PMF or SMF who underwent allo-HCT, Tamari et al. reported no implication of HMR mutations or *TP53* mutations on relapse free survival (RFS) or OS regardless of MIPSS score, however, mutations in *DNMT3A* or *U2AF1* were associated with reduced RFS and *U2AF1* also with shorter OS. Interestingly, variant allele frequencies of *JAK2V617F*, *CALR,* or *ASXL1* had no impact on RFS or OS [176]. Recently, Stevens et al. reported no implication of *ASXL1* mutations on allo-HCT outcome in an NGS study of 55 MF patients [90] in contrast to the NGS study of 169 MF patients by Kröger et al. who found a higher risk of relapse in *ASXL1* mutated patients [154]. Regarding progression free survival (PFS), only *IDH2* remained significant of the five HMR mutations, and no association with mutations in *SRSF2*, *SF3B1*, *IDH1*, *TET2*, *DNMT3A*, or *EZH2* was found. No HMR mutation had an impact on OS, while *CALR* was associated with improved OS and PFS [154]. 

Although the number of mutations (≥3 vs. <3 mutations) was not associated with RFS or OS in the study by Tamari et al. [176], a threefold higher incidence of post-HCT relapse was demonstrated in patients with ≥3 mutations in the study by Stevens and colleagues, who stressed that the discrepancy could be owing to the difference in cutoff used (including vs. excluding driver mutations, respectively) [90]. Thus, conflicting results exist regarding outcome after allo-HCT calling for larger NGS studies to address this issue.

Taken together, the clinical course of patients with ET, PV, or MF relies heavily on clinical and molecular risk factors. The mutational landscape is highly complex with a vast array of mutations influencing prognosis and disease outcome. In this regard, the multigene approach of NGS is a useful tool to identify the subgroup of patients with increased genetic instability and therefore high risk of adverse outcome. 

## 4. Use of NGS to Decipher the Mutational Landscape in MPNs in Response to Therapy 

With the advancement of high throughput technologies during the past decade, there has been a tremendous progression in the understanding of MPN disease pathogenesis. As alluded to above, a prognostic predictive molecular scoring system have been developed in ET, PV, and MF with the purpose of identifying patients at risk of poor outcome. Molecular profiling at time of diagnosis may guide treatment decisions thereby tailoring therapeutic choices complying with the heterogenic presentation of the disease. Despite these advancements, there are yet no definitive cure in patients with MPNs. Below, the most widely used treatment modalities in MPNs are highlighted in parallel with improvements in therapeutic decision-making using NGS. Treatment options in MPNs are highly divergent ranging from the “wait and watch” strategy to allogenic stem cell transplantation depending on the severity stage of the disease [135]. The principal reason for treatment in MPNs is to prevent thrombohemorrhagic complications and transformation to the advanced or blast-phase stages of the disease. 

### 4.1. Hydroxyurea

Hydroxyurea (HU) has been the choice of cytoreductive treatment for many years, however, concerns have been raised due to its mutagenic potential after long-term treatment in MPNs [215,216,217,218,219]. Conflicting results exists whether HU has any impact on the *JAK2V617F* mutational status, and it has been speculated if reduced *JAK2V617F* allele burden during treatment with HU is only a consequence of a reduction of the neutrophil cell count [220,221,222]. It is generally recognized that HU does not influence the quiescent hematopoietic stem cells, and no durable effects after treatment discontinuation have ever been observed implying that normalized cell counts will increase within days [216,223,224,225]. One targeted NGS study of HU treated MPN patients appeared in 2018, where Senin and colleagues tested a range of treatment options in ET and PV (anagrelide, busulphan, HU, P_32_, and interferon (IFN)). In patients treated with HU, the presence of additional mutations, especially in *SRSF2* or *RUNX1* at diagnosis was associated with a higher risk of developing a new mutation. Interestingly, they found no higher risk of acquiring a new mutation in patients receiving HU for more than 5 years compared with less than 5 years or no treatment. However, median follow-up was only 10 years (range 1–13) and longer follow-up time may be needed to discover any difference. In addition, they found a higher probability of cytopenia during HU in patients carrying additional mutations in *DNMT3A*, *SRSF2*, *IDH1/2* or *RUNX1* compared to patients without [164]. Resistance to HU has been associated with leukemic transformation and shorter OS, particularly in patients developing cytopenia [226], highlighting the value of NGS in guiding therapy. Indeed, studies in AML have provided evidence for a critical role of *DNMT3A* in chemotherapeutic resistance [227]. Since chemotherapeutic resistance is a major factor for drug treatment failure, it is tempting to consider how the efficacy of other treatment modalities interferes with the mutational landscape in MPNs. 

### 4.2. Interferon Alpha

The non-leukemogenic disease modifying agent interferon-alpha2 (IFN) has been used for decades in patients with MPNs [228,229,230,231,232]. Studies have convincingly demonstrated complete hematological remission within 6 months of IFN therapy in MPN patients followed by molecular remission with a reduction of the *JAK2V617F* allele burden [26,28,31,33,37,155,233,234,235,236], and in some patients even a sustained deep hematological and molecular remission together with normalization of the bone marrow after discontinuation of treatment [21,22,237]. The efficacy of IFN is likely resulting from a comprehensive range of biological properties, including boosting of virtually all immune cells, selective targeting and eventually eradication of malignant cells, and an efficient activation of dormant malignant stem and progenitor cells thereby possibly breaking their resistance to therapy [235,238,239,240,241,242,243,244,245,246,247,248,249,250,251,252,253,254]. 

In a series of 31 *CALR* mutated IFN-treated patients with ET, Verger and colleagues performed targeted sequencing and found six of 31 patients having one or more additional mutations in *ASXL1*, *IDH1/2*, *TET2,* or *TP53*. Strikingly, patients with no additional mutations had a significantly better response to IFN compared to patients presenting with ≥1 of those mutations suggesting *ASXL1*, *IDH1/2*, *TET2* or *TP53* may be associated with resistance to treatment [75]. A similar trend was observed in *ASXL1*, *DNMT3A*, *EZH2*, *IDH1/2, and TET2* in the serial single gene sequencing study of 40 ET and 43 PV *JAK2V617F* patients by Quintas-Cardama et al., however, statistical significance was not achieved [198]. Silver et al. found in their NGS study of 30 patients with MF poor response to IFN in cases with baseline mutations in *SRSF2* or *ASXL1*, although not statistically significant [157]. Interestingly, Ianotto and colleagues found non-driver mutations in 68% of patients who discontinued IFN compared to only 33% of patients who remained on IFN [160]. Finally, in a series of 202 and 135 MPNs studied at baseline and after 24 months of treatment with IFN or HU, Knudsen et al. reported that *DNMT3A* was the most frequently acquired mutation in mainly IFN treated patients not achieving clinicohematological complete response at follow-up [169]. All together, these findings strongly suggest that additional mutations may play a role in resistance to treatment with IFN. 

Quintas-Cardama et al. also showed significantly higher *JAK2V617F* response rates to IFN in *TET2* wild type patients in contrast to *TET2* mutated patients [198], whereas Kralovics et al. reported no significant difference in response rates during ropeginterferon-alpha2b in their NGS study of 163 PV patients [182]. Although studies have shown that the allele burden of *JAK2V617F* but not *TET2* decreases during IFN therapy [198,245], the allele burden of both *JAK2V617F* and *TET2* decreased significantly during treatment with ropeginterferon-alpha2b [182]. These results suggest a heterogeneous response to IFN possibly attributed to the coexistence of different clones developing independently during therapy. 

### 4.3. Ruxolitinib

Following the discovery of the *JAK2V617F* mutation in 2005, JAK1-2 inhibitor therapy was developed with ruxolitinib, a potent anti-inflammatory treatment modality, showing great benefit in reducing symptom burden and spleen size in patients with MF and PV [23,25,30,32,246,255,256,257,258] and to a lesser extent in patients with ET [259,260,261,262]. Anticipated side effects of ruxolitinib therapy such as anemia, thrombocytopenia, immunosuppression, or infections were also demonstrated in a small subset of patients [256,263,264,265]. Although ruxolitinib is not clonally selective for the malignant cells, some studies have documented a reduction of the *JAK2V617F* allele burden during treatment with ruxolitinib, whereas other studies showed only a modest reduction in the *JAK2V617F* allele burden [23,266,267,268,269,270]. In these studies, induction of remission was not experienced, however, a single exceptional case report demonstrated deep molecular remission in concert with cytogenetic remission and reversal of MF in a patient with post-PV MF [271].

In recent years, the effect of ruxolitinib on the mutational landscape in patients with MF has been elucidated using targeted NGS. In 2014, the first NGS study on the efficacy of ruxolitinib was performed by Guglielmelli et al. on 166 MF patients from the COMFORT-II trial [138]. Ruxolitinib associated spleen response and development of anemia or thrombocythemia were found unrelated to baseline HMR or LMR mutation status and to individually mutated genes [138]. Nevertheless, other studies have shown a more detrimental effect of additional mutations on the response to ruxolitinib. In their study of 95 patients with MF, Patel et al. found a lower ruxolitinib associated spleen response and shorter time to treatment discontinuation in patients with one or more mutations in *ASXL1*, *EZH2* or *IDH1/2* or with ≥3 mutations of any type [64]. Of note, *SRSF2* was not included in their targeted NGS panel [64]. Likewise, Pacilli et al. found in their study of 46 MF patients that HMR status or mutations in *ASXL1* at baseline resulted in loss of spleen response or shorter duration of spleen response, respectively after 3 years of ruxolitinib treatment, although the symptom response rate to ruxolitinib was not affected by baseline HMR mutations [163]. Furthermore, Spiegel et al. found a shorter time to treatment failure in MF patients with a HMR profile or with mutations in *ASXL1* or *EZH2*, however, they found no impact of mutations in *IDH1/2* or *SRSF2*, and no individual mutations or HMR mutations were associated with spleen response [82]. Recently, Coltro et al. performed NGS on 61 ruxolitinib treated patients with MF. After a median treatment period of 28 months, spleen response was lower in patients with mutations in the RAS/MAPK pathway genes CBL, KRAS, and NRAS [57]. Interestingly, the study by Ortmann et al. showed that the order of which *JAK2* and *TET2* was acquired influenced response to ruxolitinib with *JAK2* first having a higher sensitivity to ruxolitinib in vitro [132]. 

Acquisition of mutations or clonal expansion has been reported in regard to therapeutic management. Newberry et al. provided NGS data on 62 MF patients and found an *ASXL1* mutation at follow-up in 14 of 22 patients. Furthermore, after ruxolitinib discontinuation in 56 patients, they reported shorter OS and pretreatment transfusion dependence in patients with clonal expansion compared to patients without, although spleen response was not associated with clonal evolution [156]. Contradictory, among 46 ruxolitinib treated MF patients in the study by Pacilli and colleagues, all patients with acquisition of ≥1 mutation experienced loss of spleen response compared with 21% of patients without clonal evolution. Furthermore, acquisition of ≥1 non-driver mutation correlated with treatment discontinuation [163]. Whether the appearance of new clones are attributed to selective pressure by ruxolitinib or disease progression are a matter of debate and needs to be tested in larger studies. Taken together, accumulating evidence suggests that refractoriness to ruxolitinib may be, at least in part, attributed to additional mutations, in particular *ASXL1* and *EZH2* mutations. 

**Figure 2:**
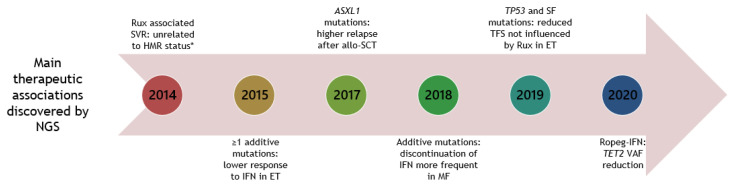
Timeline of NGS studies in patients with MPNs, unravelling main therapeutic associations. Year is shown in circular boxes. Year 2020 is until June 30. Allo-SCT: allogenic-stem cell transplantation. ET: essential thrombocythemia. HMR: high-molecular risk mutations (*ASXL1, EZH2, IDH1/2, SRSF2*). IFN: interferon-alpha. MF: myelofibrosis. Rux: ruxolitinib. SF: splice factor. SVR: spleen volume reduction. TFS: transformation free survival. VAF: variant allele frequency. * since then other studies have not been able to demonstrate this association [64,82].

In Figure 2, the main therapeutic associations discovered by NGS are depicted.

### 4.4. Combination Therapy with Interferon Alpha and Ruxolitinib

Unlike IFN therapy, ruxolitinib monotherapy does not modulate the quiescent hematopoietic stem cells [272,273]. Accordingly, a rational treatment approach may be combination therapy with ruxolitinib and IFN [274,275] enabling ruxolitinib to normalize a high-level of JAK-mediated pro-inflammatory cytokines [276], likely enhancing the capability of IFN to exert its effects by inhibiting clonal expansion and improving tumor immune surveillance [7,19,277]. In addition, as noted above, the induction of cell cycling of dormant hematopoietic stem cells by IFN mobilizes the malignant cells to targeted treatment with not only IFN itself but also e.g., JAK-inhibitors [253,254]. Adding a statin to combination therapy with IFN and ruxolitinib may have an even more profound effect on the malignant clone due to statins anti-inflammatory, antithrombotic, antiproliferative, and antiangiogenic properties [276,278]. It has been shown that statins selectively inhibit growth and viability of *JAK2V617F* MPN cells suggesting a synergistic effect with JAK-inhibitor therapy [270,279]. Indeed, the use of drug combinations has long been known to minimize therapeutic resistance [280,281]. Accordingly, studies investigating if combination therapies may counteract the adverse effects of additional mutations on treatment responses are urgently needed in the future.

### 4.5. Using NGS in Early Treatment Decisions 

Early therapeutic intervention with IFN eventually in combination with the anti-inflammatory agent ruxolitinib at the time of diagnosis, where the tumor burden is lowest, has been proposed to be essential to impair clonal evolution, subclone formation, and development of additive mutations [2,27,34,239,282] which are likely driven by chronic inflammation [2]. In fact, early treatment with a combination of IFN and HU has been suggested for a restricted time period, since their combined effects might be highly efficacious and are foreseen to have the potential to minimize the risk of thrombosis and bleeding [283]. Applying this strategy – treatment at diagnosis as in any other cancer - the progressive disease development in the biological continuum (ET-PV-MF) with increased risk of thrombosis, resistance to therapy, and leukemic transformation may hopefully be attenuated, thereby opening the avenue for patients entering minimal residual disease with deep molecular remission and normalization of the bone marrow [7,21,22,27,34,35,235,237,274,275,276,277,284]. Thus, upfront application of NGS may drive therapeutic choices, taking into account that early treatment in patients without adverse mutations may prevent development of splenomegaly, anemia, and myelofibrosis. In particular, care should be taken in patients presenting with adverse mutations, since chemotherapy may affect clonal architecture with subclone formation and appearance of new mutations in treatment resistant clones resulting in treatment failure [285]. Hopefully, combination therapy initiated at diagnosis may ameliorate treatment resistance by targeting the malignant clone before clonal expansion occurs.

### 4.6. Monitoring of Disease by NGS

The pool of genetically diverse clones has a profound effect on response to therapy. During the past decade, close monitoring of the *JAK2V617F* or *CALR* allele burdens has been performed to detect those patients achieving minimal residual disease negativity rendering them eligible for treatment cessation. However, with the advent of NGS and its increasing use in clinical practice, molecular profiling of myeloid malignancy associated genes allows for sequential monitoring of the neoplastic clones during therapy, aiming for accurate assessment of disease evolution and update choice of treatment if adverse genomic changes appear. 

## 5. Conclusions

MPNs are heterogeneous diseases not fully understood with a complex multitude of several factors such as hematological characteristics, mutational diversity, bone marrow microenvironment, stem cell biology, and clonal evolution contributing to disease pathology. Numerous phenotypes occur ranging from genuine ET to blast phase MF with quite different prognosis and outcome. In the past decade, the explosion of knowledge in genomics obtained by high-throughput sequencing has provided an abundance of useful information in patients with MPNs. Several mutational processes implicated in disease evolution, prognostication, and treatment decisions have been uncovered enabling development of molecular classification schemes and prognostic stratification models. With NGS becoming more and more implemented in clinical practice, integration of clinical data with genomic profiling data at diagnosis and during follow-up may support clinical decision-making allowing personal prediction of outcome and tailored treatment modalities for patient management. 

## Figures and Tables

**Table 1:** The most common additional gene mutations in MPNs elaborated by NGS studies.

Gene	ET %	PV %	SMF %	PMF %	Ref
**DNA methylation**					
***DNMT3A***	<10	3–15	<5	5–15	[58,59,60,61,62,63,64,65,66]
***IDH1/2***	<2	<2	<2	<5	[60,61,63,64,65,67,68]
***TET2***	10–20	15–30	20–40	10–15	[58,59,60,62,64,65,66]
**Chromatin modifiers**					
***ASXL1***	5–10	5–10	10–25	20–45	[58,59,60,61,62,63,64,65,66,67,68]
***EZH2***	<5	<5	5–15	3–12	[60,61,62,63,64,65,66,67,68]
**RNA splicing**					
***SF3B1***	<7	<6	2–14	3–18	[58,59,60,61,62,63,65,69]
***SRSF2***	<5	<5	<5	10–35	[58,59,60,61,62,63,65,66,67,68]
***ZRSR2***	<3	<5	5–10	1–10	[59,60,61,62,63]
***U2AF1***	<5	<5	5–20	5–20	[58,59,60,61,62,63,65,66]
**Signaling**					
***CBL***	<7	<7	<5	<7	[57,58,60,61,65]
***KIT***	2	3	NA	<2	[59,60]
***NRAS***	<2	<2	<3	<5	[57,59,60,61,63,64,65]
***SH2B3***	3–5	<9	5	6	[60,61]
**Transcription factors**					
***CEBPA***	4	2–6	NA	9	[59,60,63]
***RUNX1***	<3	<2	<3	<5	[59,60,61,63,64,65]
**Tumor suppressors**					
***TP53***	<9	<5	<14	<7	[58,59,60,61,62,63,64,65,66]
**DNA damage**					
***PPM1D***	3	1	<1	<1	[20]

ET: essential thrombocythemia. PMF: primary myelofibrosis. PV: polycythemia vera. SMF: secondary myelofibrosis.

**Table 2:** NGS studies in patients with MPNs.

Study (Author)	Patients (No) and Disease	Type of Study	Method (Gen No)	Main Prognostic Findings	Ref
Hou et al., 2012	1 ET	Baseline single cell	Illumina (WES)	NA	[122]
Klampfl et al., 2013	6 PMF	Baseline	Illumina (WES)	NA	[12]
Merker et al., 2013	WGS: 1 PMF Targeted seq: 40 ET, 42 PV, 96 MF	Baseline	CompleteGenomics (WGS), Illumina (6)	NA	[141]
Nangalia et al., 2013	62 ET, 48 PV, 39 PMF, 2 MPN-U	Baseline and follow-up	Illumina (WES)	*CALR* mut ET higher risk of MF	[13]
Guglielmelli et al., 2014	27 PET-MF, 54 PPV-MF, 85 PMF	Baseline and Rux	Roche 454 GS or PGM (14)	Spleen response unrelated to HMR or LMR mutations during rux	[138]
Lundberg et al., 2014	69 ET, 94 PV, 34 PMF	Serial follow-up	Illumina (104)	*TET2*, *TP53*, ≥2 mutations: shorter OS, LFS	[120]
Tenedini et al., 2014	Study 1: 9 PV, 5 PPV-MF, 11 PMF Validation:50 PV, 48 SMF, 91 PMF	Baseline and follow-up	Roche 454 GS (WES), PGM (121)	*NRAS* in PMF: shorter OS	[142]
Wang et al., 2014	31 PV	Baseline	Illumina (WES) and PGM (42)	NA	[143]
Angona et al., 2015	36 PV, 9 PPV-MF	Baseline	Roche 454 GS (4)	NA	[144]
Engle et al., 2015	1 PMF	Baseline and follow-up	Illumina WGS, targeted seq (58)	NA	[123]
Kirschner et al., 2015	10 ET, 9 SMF, 17 PV, 10 PMF	Baseline	Illumina (48)	NA	[145]
Ortmann et al., 2015	First cohort: 92 ET, 107 PV, 47 MF Follow-up cohort: 918 MPN	Baseline and Rux	Exome or Illumina (111/65)	*JAK2* first vs. *TET2* first: younger, higher risk of thrombosis	[132]
Patel et al., 2015	10 PET-MF, 31 PPV-MF, 54 PMF	Baseline and Rux	Illumina (28)	≥1 mutations in *ASXL1*, *EZH2*, *IDH1/2*: shorter OS and TTD during rux.	[64]
Verger et al., 2015	31 ET, Control group: 12 ET(aspirin only), 14 ET–HU only	Baseline and IFN	Illumina (7)	≥1 additional mutation: higher rate of no response to IFN	[75]
Angona et al., 2016	29 ET, all triple negative	Baseline	Roche 454 GS (NA)	NA	[73]
Asp et al., 2016	ET: 8 TN, 18 CALR, 18 JAK2V617F, 7 MPL. PMF: 7 TN, 1 MPL	Baseline and follow-up	Illumina (54)	TN (PMF), *MPL* (ET): shorter OS *ASXL1*, *SRSF2* in ET: shorter OS	[146]
Cabagnols et al., 2016	17 ET, all triple negative	Baseline	Illumina (WES)	NA	[147]
Delic et al., 2016	40 ET, 30 PV, 30 PMF	Baseline	Illumina (28)	*ASXL1*, *EZH2*, *SF3B1*, *SRSF2*, *U2AF1* more mutated in PMF than ET	[58]
Jeromin et al.,2016	88 MPN	Baseline	Roche 454 GS (1)	NA	[148]
Magor et al., 2016	16 ET, 8 PV, 6 SMF, 11 PMF	Baseline	Ion torrent PGM (86)	NA	[149]
M Feenstra et al., 2016	4 ET, 4 PMF	Baseline	Illumina WES	NA	[150]
Rotunno et al., 2016	165 PET-MF, 194 PPV-MF	Baseline and follow-up	PGM (5)	TN, *SRSF2* (PET-MF): shorter OS	[67]
Tefferi et al., 2016	Mayo cohort: 183 ET, 133 PV Italian cohort: 174 ET, 215 PV	Baseline and follow-up	PGM (5), Illumina (27)	PV: *SRSF2*: shorter OS, LFS, MFS ET: *IDH2*, *SH2B3*: shorter OS.	[59]
Tefferi et al., 2016	182 PMF	Baseline and follow-up	PGM (5), Illumina (27)	*ASXL1*, *SRSF2*, *CBL*, *KIT:* shorter OS *SRSF2, RUNX1*, *SH2B3*, *CEBPA*: shorter LFS	[60]
Agarwal et al., 2017	114 ET, 3 PET-MF, 5 PPV-MF, 44 PMF	Baseline	Illumina (26)	*CALR* type 1: more common in PMF than *CALR* type 2	[66]
Casolari et al., 2017	15 PV	Baseline	Solid (657)	NA	[151]
Chang et al., 2017	7 ET, 8 PV, 1 PMF, all triple negative	Baseline	Ion Proton (409)	NA	[152]
Courtier et al., 2017	57 Chronic phase MPN, 38 Post-MPN AML	NGS in chronic phase during disease	Illumina (79)	Acute phase an average gain of 1 mutation compared to chronic phase.	[153]
Kröger et al., 2017	101 PMF, 46 SMF, 13 MF transformed	Baseline and follow-up	Solid and PGM (5 and 18)	*ASXL1*: relapse. *IDH2*: worse PFS. *CALR*: improved PFS and OS	[154]
Luque Paz et al., 2017	22 ET, 28 PV, 50% selected with disease progression	Baseline and follow-up after 3 years	PGM (18)	≥2 mutations or *ASXL1*, *IDH1/2*, or *SRSF2*: disease progression	[86]
Masarova et al., 2017	6 ET/PV	Baseline and IFN	NA (44)	*DNMT3A*, *ASXL1* acquired at transformation	[155]
Newberry et al., 2017	62 MF	Baseline and Rux	Illumina (28)	Clonal evolution after Rux: shorter OS	[156]
Silver et al., 2017	2 PET-MF, 7 PPV-MF, 21 PMF	Baseline and IFN	Illumina (45)	≥3 mut, *ASXL1*, *SRSR2*: adverse events	[157]
Song et al., 2017	27 ET, 33 PV, 75 PMF	Baseline	Illumina (32)	ASXL1, *SRSF2* more frequent in PMF	[85]
Spiegel et al., 2017	23 PET-MF, 27 PPV-MF, 50 PMF	Baseline and Rux and MMB	Illumina (54)	≥3 mut, HMR, *ASXL1*, *EZH2*: shorter OS	[82]
Zaidi et al., 2017	1 ET	Baseline and follow-up	Illumina (54)	NA	[158]
Alduaij et al., 2018	21 ET, 26 PV, 28 PET-MF, 15 PPV-MF, 64 PMF, 12 Post-MPN-AML	Baseline and follow-up	Illumina (54)	HMR mutations versus no HMR mutations: early HCT vs. delayed HCT	[65]
Ayres-Silva et al., 2018	3 ET and 3 paired Post-ET-AML	Baseline and transformation	Illumina (WES)	TP53 mutations during transformation, all HU	[159]
Bartels et al., 2018	36 PV with stable disease, 28 PV with fibrotic progression	Baseline and follow-up	NA (23)	Additional mutations (not *TET2*): increased risk of fibrotic progression	[89]
Grinfeld et al., 2018	1321 ET,356 PV,309 MF,14 MPN-U, 35 other. Serial:290 ET,30PV,10MF	Baseline and follow-up	Illumina (69 and WES)	Developed a prognostic classification system	[20]
Guglielmelli et al., 2018	490 PMF and 315 PMF	Baseline and follow-up	PGM 5, Illumina 27	≥1 HMR mutations: shorter OS	[68]
Ianotto et al., 2018	49 MF	Baseline and IFN	PGM (26)	≥1 mutation: shorter LFS	[160]
Ju et al., 2018	68 ET	Baseline	NA (360)	NA	[161]
Kubesova et al., 2018	Untreated: 22 ET, 22 PV, 36 PMF Treated: 80 ET, 116 PV, 53 PMF	Baseline and follow-up (HU, IFN, ANA)	Illumina (1)	*TP53* mutations are associated with age	[162]
Pacilli et al., 2018	Rux: 7 PET-MF, 16 PPV-MF, 23 PMF. HU: 6 SMF, 19 PMF	Baseline and Rux or HU	PGM (27)	*ASXL1*: shorter duration of spleen volume reduction	[163]
Senin et al., 2018	Baseline: 37 ET, 63 PV. Follow-up: 50 No progression, 24 MF, 12 AML	Baseline and ANA, BUS, P3S2, IFN, HU	Illumina and Roche 454 GS (50)	*SF3B1*, *IDH1/2*: higher MT. *ASXL1*, *TP53*, *SRSF2*, *IDH1/2*, *RUNX1*: higher LT	[164]
Tefferi et al., 2018	641 PMF	Baseline and follow-up	PGM 5, Illumina 27	*ASXL1*, *SRSF2*: shorter OS, LFS	[165]
Tefferi et al., 2018	100 MF	Baseline and MMB	Illumina (27)	*ASXL1*, *SRSF2*: shorter OS, *SRSF2* shorter LFS	[166]
Acha et al., 2019	35 ET (TN), 8 PMF (TN)	Baseline and follow-up	Illumina (17)	≥1 mutation: shorter OS	[167]
Beucher et al., 2019	1 triple negative ET with rare JAK2 and MPL mutations	Baseline and HU	Illumina (69)	*SF3B1* VAF increased and *TET2*, *JAK2*, *MPL* decreased during HU	[101]
Boiocchi et al., 2019	29 ET, 21 PV, 51 PMF, 21 SMF, 21 MPNU	Baseline	Illumina (101)	MPN: *SF3B1*: lower hemoglobin	[69]
Byun et al., 2019	16 ET, 17 PV, 8 PMF	Baseline and follow-up	Illumina (47)	*ASXL1*: higher risk of LT. Splicing gene mutations: shorter OS, higher LT	[87]
Courtier et al., 2019	31 PET-MF, 28 PPV-MF, 86 PMF	Baseline and follow-up	Illumina (79–106)	SMF: *ASXL1*, *TP53*: shorter OS. PMF: *SRSF2*, *TP53*: shorter OS	[62]
Gagelmann et al., 2019	55 PET-MF, 46 PPV-MF, 260 PMF	Baseline and HCT	Solid and PGM (20)	*ASXL1*: shorter OS after HCT	[168]
Gill et al., 2019	17 PET-MF, 14 PPV-MF, 70 PMF	Baseline and follow-up	Illumina (54)	SMF + PMF: *CUX1*, *TP53*: shorter OS. *SRSF2*: shorter LFS	[88]
Knudsen et al., 2019	72 ET, 89 PV, 16 Pre-PMF, 25 PMF	Baseline and IFN, HU	Illumina (100)	*DNMT3A* acquired during IFN	[169]
Luque Paz et al., 2019	1190 ET	Baseline and follow-up	Illumina (16)	≥1 mutation: shorter OS	[170]
Mannina et al., 2019	14 PMF, 4 PET-MF (all MPL positive)	Baseline and HCT	PGM (20)	*MPL:* favorable OS after HCT	[171]
Nam et al., 2019	6 ET, 5 MF	Baseline	Illumina (45)	NA	[172]
O’Sullivan et al., 2019	110 ET	Baseline and Rux	Illumina (32)	*SF3B1*, *TP53*: shorter TRFS	[173]
Rodriguez-Meira et al., 2019	1 ET, 1 PV, 3 SMF, 5 PMF	Baseline	Illumina single cell	NA	[174]
Schischlik et al., 2019	30 ET, 1 PV, 46 PMF	Baseline	Illumina (54)	NA	[175]
Stengel et al., 2019	50 CALRpos	Baseline	Illumina (14)	*SF3B1* associated with CN-LOHpos *TP53* associated with del5q.	[91]
Szuber et al., 2019	PMF (99–120 patients)	Baseline and follow-up	NA (6)	≥1 HMR: shorter LFS, OS, TFS	[83]
Tamari et al., 2019	62 PMF, 20 Post-ET-MF, 18 Post-PV-MF, 1 MPN-U	Baseline and follow-up (HCT)	NA (585)	*DNMT3A*, *U2AF1*: shorter RFS. HMR mutations: no impact on OS or RFS	[176]
Wanquet et al., 2019	35 ET, 14 PV, 31 PET-MF, 28 PPV-MF. Paired: 2 ET, 6 PV	Baseline and follow-up	Illumina (33)	TP53: shorter OS	[61]
Yacoub et al., 2019	110 ET/PV	Baseline + IFN	Illumina (156)	CALR mutation: higher CR	[177]
Andreasson et al., 2020	85 PV	Baseline and follow-up	Illumina (54)	*ASXL1,* vascular complication, ≥3 mutations: shorter OS	[63]
Bartels et al., 2020	PMF without (27) or with (77) development of fibrosis	Baseline and follow-up	PGM (23)	*SRSF2*, *U2AF1*, *SF3B1*, *IDH1/2*, *EZH2* risk factors for fibrotic progression	[84]
Cassinat et al., 2020	233 ET, 187 PV, 169 MF	Baseline	Illumina (36)	NA	[178]
Coltro et al., 2020	132 PF-PMF, 155 PMF, 177 SMF	Baseline and Rux		*CBL*, *KRAS*, *NRAS*: shorter OS and LFS	[57]
Cottin et al., 2020	45 ET	Baseline and follow-up	Illumina (52)	8 *TET2* and 1 *DNMT3A*: higher VAF at follow-up	[77]
Gill et al., 2020	56 ET, 23 PV, 46 MF	Baseline, IFN, HU, Rux	Illumina (69)	PV: CREBBP: inferior response rate	[179]
Guglielmelli et al., 2020	132 pre-PMF	Baseline and follow-up	PGM (5)	HMR: associated with arterial thrombosis	[180]
Karantanos et al., 2020	66 ET, 31 PV, 64 PMF, 49 SMF, 9 AML	Baseline and follow-up	NA (63)	Higher number of additional mutations in men compared to women	[181]
Kralovics et al., 2020	163 PV	Baseline and IFN	Illumina (54)	*JAK2*, *TET2* decrease during ropeg-IFN	[182]
Mylonas et al., 2020	WES: 8 PMF, 7 PET/PPV-MF Targeted seq: 7 MF	Baseline and Rux	Illumina (WES) and targeted seq	Mutations were acquired in *BRAF*, *CBL*, *KRAS*, *NRAS*, and *RIT1*	[183]
Nonino et al., 2020	27 MF	Baseline	Illumina (255)	NA	[184]
Segura-Diaz et al., 2020	25 ET, 16 PV, 16 PMF, 11 SMF and PV case-control cohort (55)	Baseline and follow-up	Illumina (30)	DTA mutations: associated with vascular events in PV	[185]
Stevens et al., 2020	22 PMF, 33 SMF	Pre and post-HCT	Illumina (75)	≥3 additional mutations pre-transplant: higher PTR and NRM	[90]
Tefferi et al., 2020	502 ET, 404 PV	Baseline and follow-up	PGM (5), Illumina (27)	ET: *SF3B1*, *SRSF2*, *EZH2*: shorter OS PV: *SRSF2*, *IDH2*: shorter OS	[186]

ANA: anagrelide. BUS: busulfan. CN-LOH: Copy neutral-loss of heterozygosity. CR: complete remission. DTA: DNMT3A, TET2, ASXL1. ET: essential thrombocythemia. HCT: hematopoietic stem cell transplantation. HMR: high-molecular risk mutations. HU: hydroxyurea. IFN: interferon-alpha. LFS: leukemia-free survival. LT: leukemic transformation. MF: myelofibrosis. MFS: myelofibrosis-free survival. MMB: momelotinib. MT: myelofibrotic transformation. NRM: non-relapse mortality. OS: overall survival. Pre-PMF: prefibrotic-PMF. PFS: progression free survival. PGM: Ion Torrent Personal Machine. PMF: primary myelofibrosis. PTR: post transplant relapse. PV: polycythemia vera. RFS: relapse free survival. Rux: ruxolitinib. SMF: secondary MF. TFS: thrombosis free survival. TN: triple negative. TRFS: transformation free survival. TTD: time to treatment discontinuation. TTF: time to treatment failure. WES: whole exome sequencing. VAF: variant allele frequency. WGS: whole genome sequencing.

**Table 3:** The clinical significance of the most common additional gene mutations in MPNs elaborated by NGS studies.

Gene	ET	PV	SMF	PMF	Comments	Ref
**DNA methylation**						
***DNMT3A***	Risk of HU-cytopenia.	Higher risk of MT. Higher risk of HU-cytopenia	Reduced RFS after HCT.	Reduced RFS after HCT.		[89,164,176]
***IDH1/2***	Shorter OS (IDH2). Higher risk of MT, LT. Higher risk of HU-cytopenia.	Shorter OS (IDH2). Higher risk of MT, LT. Higher risk of HU-cytopenia.	Shorter OS. Higher risk of LT. Lower SVR during Rux. Worse PFS after HCT (*IDH2*).	Shorter OS. Higher risk of LT. Lower SVR during Rux. Worse PFS after HCT (*IDH2*).		[59,64,86,89,154,164,186]
***TET2***	Associated with older age and thrombosis.	Associated with thrombosis.	NA	Associated with older age.	Shorter OS and higher LT in MPNs	[59,60,120,185]
**Chromatin modifiers**						
***ASXL1***	Shorter OS. Higher risk of LT. Associated with splenomegaly.	Shorter OS. Higher risk of LT. Associated with thrombosis and older age.	Shorter OS. Higher risk of LT. Shorter TTF and duration of SVR during Rux. Higher relapse and shorter OS after HCT.	Shorter OS. Higher risk of LT. Shorter TTF and duration of SVR during Rux. Higher relapse and shorter OS after HCT.	Lower Hb in MPNs.	[59,60,62,63,64,82,86,120,147,154,155,164,165,166,169]
***EZH2***	Shorter OS. Higher risk of LT, MT.	NA	Shorter OS. Higher risk of LT. Shorter TTF during Rux.	Shorter OS. Higher risk of LT. Shorter TTF during Rux.	Higher leukocyte counts in MPNs.	[59,61,64,82,120,186]
**RNA splicing**						
***SF3B1***	Shorter OS. Higher risk of MT, LT. Higher platelets.	Higher risk of MT.	NA	NA	Lower Hb in MPNs.	[59,69,164,186]
***SRSF2***	Shorter OS. Higher risk of LT. Higher risk of HU-cytopenia.	Shorter OS. Higher risk of MT, LT. Higher risk of HU-cytopenia.	Shorter OS.	Shorter OS. Higher risk of LT. Associated with anemia.		[60,67,146,164,165,186]
***ZRSR2***	NA	NA	Worse prognosis	NA		[61,62]
***U2AF1***	Higher risk of MT.	Higher risk of MT.	Reduced RFS and OS after HCT.	Shorter OS. Associated with anemia and thrombocythemia. Reduced RFS, OS after HCT.		[59,60,165,176,186]
**Signaling**						
***CBL***	NA	NA	Shorter TTF during Rux.	Shorter OS. Shorter TTF during Rux.		[60,82]
***KIT***	NA	NA	NA	Shorter OS.		[60]
***NRAS***	NA	NA	Higher risk of LT.	Higher risk of MT, LT.		[142,183]
***SH2B3***	Shorter OS.	Associated with splenomegaly.	NA	Higher risk of LT.		[59,60]
**Transcription factors**						
***CEBPA***	NA	NA	NA	Higher risk of LT		[60]
***RUNX1***	Higher risk of LT. Higher risk of HU-cytopenia.	Higher risk of LT. Higher risk of HU-cytopenia.	NA	Higher risk of LT		[60,164,186]
**Tumor suppressors**						
***TP53***	Higher risk of LT.	Higher risk of LT.	Shorter OS. Higher risk of LT.	Shorter OS. Higher risk of LT.	Shorter OS and higher LT in MPNs	[59,61,62,120,153,164,186]
**DNA damage**						
***PPM1D***	Unclear	Unclear	Unclear	Unclear		[20]

ET: essential thrombocythemia. Hb: hemoglobin. HCT: hematopoietic stem cell transplantation. HU: hydroxyurea. HU-cytopenia: cytopenia during HU treatment. LT: leukemic transformation. MF: myelofibrosis. MT: myelofibrotic transformation. OS: overall survival. PFS: progression free survival. PMF: primary myelofibrosis. PV: polycythemia vera. RFS: relapse free survival. Rux: ruxolitinib. SMF: secondary MF. SVR: spleen volume reduction. TTF: time to treatment failure.
